# The effect of exercise interventions on enhancing psychological resilience: a systematic review and meta-analysis

**DOI:** 10.1186/s40359-026-04940-5

**Published:** 2026-06-06

**Authors:** Cong Liu, Jinqi Liang, Yuyang Nie, Wenxue Ma, Wenfeng Tan, Jincheng Zhong, Zixiang Xia, Ziyu Fan, Qin Qin

**Affiliations:** 1 College of Education for the Future, Beijing Normal University, Zhuhai, China; 2 College of Physical Education and Sports, Beijing Normal University, Beijing, China; 3 School of Educational Science, Huizhou University, Huizhou, China; 4 Institute for Educational Development of the Guangdong-Hong Kong-Macao Greater Bay Area, South China Normal University, GuangZhou, China; 5 School of Journalism and Communication, Beijing Sport University, Beijing, China

**Keywords:** Exercise intervention, Psychological resilience, Meta-analysis, Moderating factors, Intervention duration

## Abstract

**Background and objective:**

Amid increasing social pressures, the importance of psychological resilience as a key resource for stress coping has become prominent. Although exercise is considered a potential means to enhance resilience, a systematic quantitative assessment of moderating factors influencing its effect is lacking. This meta-analysis aimed to systematically evaluate the effect of exercise interventions on enhancing psychological resilience and explore the moderating roles of intervention duration, age, and study design.

**Methods:**

Following PRISMA guidelines, relevant databases were searched up to January 2026. Randomized controlled trials or quasi-experimental studies were included. Study quality was assessed using standardized tools. Analyses were conducted using a random-effects model, subgroup analyses, and heterogeneity tests.

**Results:**

Ten studies were included. Exercise intervention had a significant positive effect on psychological resilience (pooled SMD = 0.49, 95% CI: 0.33–0.65). Intervention duration was a statistically significant moderator (p = 0.01) that survived Bonferroni correction for multiple subgroup tests (α < sub > adjusted < /sub > = 0.017), with long-term interventions (> 8 weeks, SMD = 0.64) yielding significantly larger effects than short-term interventions (≤ 8 weeks, SMD = 0.30). The intervention was effective across all age groups. Randomized controlled trials yielded a numerically larger effect size (SMD = 0.56) than non-randomized studies (SMD = 0.35), but the subgroup difference was not statistically significant (p = 0.21). No publication bias was detected.

**Conclusion:**

Exercise intervention is an effective approach to enhancing psychological resilience. Subgroup analyses confirmed that intervention duration is a statistically significant moderator, with long-term interventions yielding significantly greater benefits. This provides an empirical basis for designing personalized, long-term exercise programs for different populations.

**Supplementary Information:**

The online version contains supplementary material available at 10.1186/s40359-026-04940-5.

## Introduction

In contemporary society, psychological resilience has emerged as a pivotal resource for individuals coping with stress, adversity, and challenges, playing a central role in maintaining mental health and enhancing quality of life [1]. Globally, mental disorders affect over 1 billion people – 14% of the population – with anxiety and depression accounting for more than two-thirds of cases. Between 2011 and 2021, the age-standardized prevalence increased by 0.9%, with the largest rise (1.8%) among young adults aged 20–29 years [2]. These figures underscore the urgent need for effective interventions, such as exercise, to enhance psychological resilience. Enhancing resilience is not only essential for personal development but also a critical public health concern, particularly among adolescents, older adults, and individuals with chronic conditions [3]. Physical activity, characterized by its high accessibility and cost-effectiveness, is widely regarded as an effective avenue for promoting mental health and bolstering psychological resilience. However, translating this potential into evidence-based, generalizable, and efficacious intervention programs remains a key scientific challenge. Core to this challenge is elucidating the moderating factors underlying the effects of exercise interventions, such as intervention duration, participant age characteristics, and the study design itself.

Psychological resilience is widely conceptualized as a dynamic and malleable psychological process, involving the effective integration of cognitive, emotional, and behavioral resources during an individual's interaction with their environment [4]. In the field of sport and health psychology, a substantial body of empirical research has corroborated a positive association between regular physical activity and higher levels of psychological resilience [5, 6]. The underlying mechanisms are multifaceted, encompassing the enhancement of self-efficacy and satisfaction of basic psychological needs [7], improvements in executive function and emotion regulation capacity [8], and mediation through pathways such as elevated self-esteem and increased social support [9]. For specific populations, such as cancer patients, adolescents, and older adults, structured exercise interventions—including aerobic training and mind–body integration practices—have demonstrated significant efficacy in enhancing psychological resilience [3, 10–12]. Furthermore, integrated interventions combining exercise with cognitive-behavioral therapy or mindfulness training have broadened the scope of resilience-promoting strategies [13, 14]. Collectively, these studies provide a foundational framework for understanding how exercise fosters resilience and highlight the value of interdisciplinary approaches in promoting health and well-being across the lifespan [15].

Despite the prevailing evidence supporting the positive impact of exercise on psychological resilience, significant limitations and knowledge gaps persist in this field of research. First, existing research often adopts a narrow focus, either concentrating on specific types of physical activity or being confined to particular populations (e.g., athletes), thereby lacking systematic comparisons and evaluations of the effects of different exercise intervention programs [16]. Second, the absence of a consensus on the definition and measurement of psychological resilience introduces theoretical inconsistencies. This conceptual ambiguity contributes to heterogeneity across studies, complicating the direct comparison and synthesis of findings [1]. Particularly critical is the insufficient and unsystematic examination of key variables that may significantly moderate the effects of exercise interventions. These variables include intervention duration and intensity, participant age and developmental stage, and the type of study design employed. For instance, it remains unclear whether the effects differ between short-term and long-term interventions, whether the same intervention yields consistent effects across children, adolescents, and older adults, and whether different study designs introduce systematic bias in effect size estimations. The inadequate understanding of these moderating factors severely constrains the precise optimization and personalized implementation of exercise interventions and impedes the advancement of relevant theoretical models.

Although exercise interventions are recognized for enhancing psychological resilience, the moderating effects of key factors—intervention duration, participant age, and study design—remain insufficiently examined. Intervention duration may influence the magnitude and sustainability of effects, with longer programs potentially yielding greater benefits [17]. Age is critical because developmental stages affect cognitive and emotional responses to exercise [15]. Study design impacts internal validity and effect size estimates, as randomized controlled trials provide more robust evidence than quasi-experimental designs [18]. Accordingly, this meta-analysis systematically investigates these moderators to guide the development of targeted exercise interventions.

To address these knowledge gaps, this study aims to conduct a systematic review and meta-analysis to comprehensively evaluate the overall effect of exercise interventions on enhancing psychological resilience, with a specific focus on investigating the moderating roles of key factors, including intervention duration, participant age, and study design. The specific objectives are: (1) to quantify the overall effect size of exercise interventions on psychological resilience enhancement; (2) to examine, via subgroup analysis and meta-regression, whether intervention duration, participant age group, and study design type are significant moderators; and (3) to provide an empirical basis for developing more targeted and personalized exercise intervention programs based on the findings. We hypothesize that exercise interventions have a positive effect on promoting psychological resilience and that this effect is significantly influenced by the aforementioned moderating variables.

## Methods

### Literature search strategy

A structured electronic literature search was conducted following the Preferred Reporting Items for Systematic Reviews and Meta-Analyses (PRISMA) guidelines. The search was executed by the first author on January 7, 2026, across five databases: Web of Science, PubMed, ProQuest, Scopus, and EBSCO. The search strategy was: [Title/Abstract] = ("physical activit" OR exercise OR "physical training" OR sport* OR "resistance training" OR "aerobic training" OR "motor activity" OR workout* OR "fitness training") AND [Title/Abstract] = ("psychologic* resilience" OR resilience OR "mental resilience" OR "emotional resilience" OR resilient OR "mental toughness" OR "coping ability" OR hardiness OR grit) AND [Title/Abstract] = (interven* OR trial* OR program* OR train* OR therap* OR treat* OR rehabilitat*). The search was limited to peer-reviewed articles published in English or Chinese, with a publication date range from January 1, 2000, to January 7, 2026.

### Protocol and registration

This systematic review and meta-analysis were conducted following the PRISMA guidelines. The review protocol was retrospectively registered with PROSPERO (CRD420261293052) on 25 January 2026, after the initial literature search (7 January 2026). All planned analyses were prespecified in the protocol, and no deviations occurred.

### Inclusion and exclusion criteria

The inclusion criteria were as follows: 1. Studies involving participants of any age. 2. Studies that assessed psychological resilience using a validated standardized instrument (e.g., the Connor-Davidson Resilience Scale, Brief Resilience Scale, or Resilience Scale). 3. Studies implementing a structured, planned physical activity program (e.g., aerobic training, resistance training, school-based yoga emphasizing physical postures, or high-intensity interval training). Programs in which meditation, mindfulness, or breathing exercises constituted the core component, with physical movement serving only as a vehicle, were excluded. Interventions combining exercise with substantial psychological components were initially included, with sensitivity analyses planned to isolate the effect of exercise alone. 4. Randomized controlled trials (RCTs) or quasi-experimental studies that included a comparator condition. Eligible comparators were no-intervention control, wait-list control, or usual care; single-group pre-post studies without any comparator were excluded from the primary analysis. 5. Studies reporting psychological resilience outcomes assessed using standardized tools, with data available for effect size calculation.

Studies were excluded if they: 1. Were not peer-reviewed. 2. Did not provide data necessary for analysis. 3. Were reviews, commentaries, or secondary analyses.

After duplicate removal, two reviewers independently screened the titles and abstracts of all retrieved records based on predefined inclusion and exclusion criteria. Full texts of potentially eligible articles were then retrieved and assessed independently. Additionally, the reference lists of included studies and relevant systematic reviews were manually searched to identify further eligible reports. Inter-rater reliability for study selection, assessed using Cohen’s kappa coefficient, was 0.89, indicating almost perfect agreement. Any disagreements during the screening or quality assessment phases were resolved through discussion or, if necessary, adjudication by a third reviewer. The final set of included studies was confirmed after cross-verification by both reviewers.

### Data extraction

Data were extracted independently by the authors using a standardized template, and any disagreements were resolved through consensus. The extracted information included: (1) Author(s) and publication year; (2) Study design; (3) Study location; (4) Sample size; (5) Participant age; (6) Specific details of the intervention; (7) Measurement tool(s) for psychological resilience; (8) Key data for calculating effect sizes; (9) Study conclusions.

### Assessment of methodological quality

The methodological quality of included studies was assessed using the checklist developed by Downs and Black [18]. This tool was selected because it is one of the few validated instruments specifically designed to evaluate both randomized and non-randomized intervention studies—a methodological feature directly relevant to this review's inclusion criteria. Although originally published in 1998, the checklist has been widely adopted in contemporary systematic reviews and meta-analyses across health and behavioral sciences, with subsequent empirical studies confirming its reliability and validity for assessing diverse study designs [19]. The tool comprises 27 items across five domains: reporting, external validity, internal validity (bias), internal validity (confounding), and statistical power. Scoring followed the original authors' guidelines, with total scores ranging from 0 to 32. Two reviewers independently rated each study, and discrepancies were resolved through discussion or third-reviewer adjudication. We acknowledge that more recent risk-of-bias tools (e.g., ROBINS-I) exist,however, the Downs and Black checklist remains appropriate for reviews combining RCTs and quasi-experimental evidence.

### Statistical analysis

The included studies primarily reported data on the effectiveness of exercise interventions on psychological resilience across populations, along with respective sample sizes. Given the use of different instruments to measure psychological resilience (e.g., Connor-Davidson Resilience Scale, Mental Toughness Questionnaire) across studies, the standardized mean difference (SMD) was selected as the summary effect size metric to pool the results. Specifically, for studies with a parallel control group, Cohen’s d was calculated using post-intervention data. For single-group pre-post studies, Cohen's d was calculated from the mean change assuming a pre-post correlation of 0.70, based on typical test–retest reliabilities of resilience scales [20] and meta-analytic guidelines [21].

Each intervention arm in multi-arm studies was analyzed separately. When a non-exercise comparator with complete post-intervention data was available, between-group Cohen's d was calculated using post-intervention scores (e.g., [22], where post-test data from the control group were used for a between-group comparison). The pre-post method was applied only when: (a) all arms consisted of active exercise interventions with no non-exercise comparator [23, 24],or (b) the comparator was an active control condition rather than standard care or no treatment [25].

The meta-analysis was performed using STATA software (version 19.0). A random-effects model was employed to pool effect sizes and compute their 95% confidence intervals (CIs). All statistical significance was set at *p* < 0.05 (two-tailed). Potential moderators were explored through subgroup analyses. First, to examine effects across different populations, subgroups were defined as minors (< 18 years), adults (18–60 years), and older adults (> 60 years). Second, a subgroup analysis by study design (randomized controlled trials [RCTs] vs. non-randomized controlled trials [NRCTs]) was conducted. Third, to assess the effect of intervention duration, interventions lasting ≤ 8 weeks were classified as short-term, and those lasting > 8 weeks as long-term [26], followed by a subgroup analysis. As three subgroup moderator tests were performed (intervention duration, age group, study design), the significance threshold for subgroup difference tests was adjusted using the Bonferroni method to control the family-wise error rate. The adjusted threshold was α < sub > adjusted </sub > = 0.05/3 ≈ 0.017. Subgroup differences with nominal *p* < 0.017 were considered statistically significant after correction. Between-study heterogeneity was assessed using the I^2^ statistic and the Q test. Potential publication bias was evaluated by visual inspection of funnel plots and quantitatively using Egger’s test for small-study effects.

### Sensitivity analyses

To test the robustness of the pooled effect size, we performed a leave-one-out analysis by iteratively removing one study at a time and recalculating the overall effect. Additional sensitivity analyses were conducted to address specific methodological concerns: (1) excluding studies that combined exercise with psychological components, (2) restricting to studies using the CD-RISC scale, and (3) varying the assumed pre-post correlation coefficient from 0.50 to 0.90. All sensitivity analyses used the same random-effects REML model.

## Results

### Study selection process

**Fig. 1:**
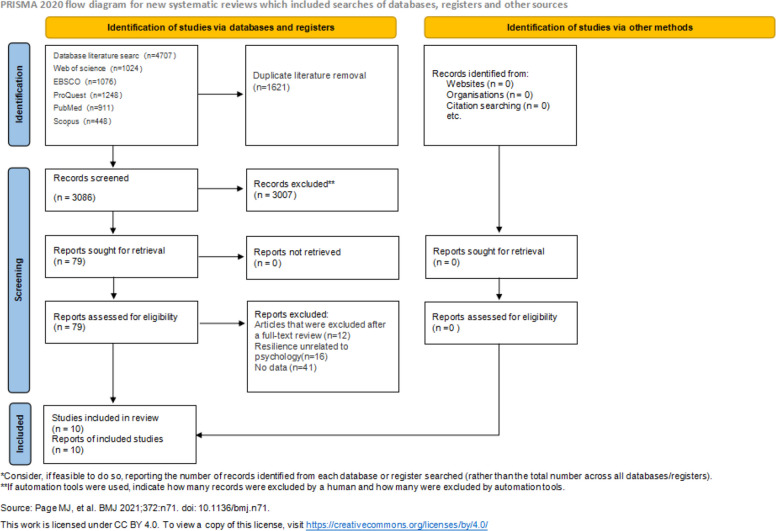
PRISMA flow diagram of the study selection process

The initial database search yielded 4707 records from the following sources: Web of Science (*n* = 1024), EBSCO (*n* = 1076), ProQuest (*n* = 1248), PubMed (*n* = 911), and Scopus (*n* = 448). After removing 1621 duplicates, 3086 unique records underwent title and abstract screening. At this stage, 3007 records were excluded as irrelevant or not meeting the inclusion criteria, leaving 79 articles for full-text review. Following a detailed full-text assessment, 69 articles were further excluded. Reasons for exclusion included: studies where the resilience concept was non-psychological (*n* = 16) and studies with unavailable or incomplete data (*n* = 41). An additional 12 articles were excluded during full-text screening for not meeting other eligibility criteria related to study design, population, or intervention. Ultimately, 10 articles met all inclusion criteria and were included in the qualitative synthesis and meta-analysis of this systematic review. The flow of study selection is presented in Fig. 1.

### Characteristics of included studies

A total of 10 intervention studies were included, all employing either randomized controlled trial (RCT) or quasi-experimental designs. Participants spanned three distinct age groups: older adults, adults, and minors, with sample sizes ranging from 30 to 701. The interventions were diverse, including online-supervised exercise, structured physical activity, psychological intervention combined with rehabilitation exercise, selective exercise training, high-intensity interval training, mindful yoga, virtual exercise training, and Body Pump training, among others. Psychological resilience was primarily measured using tools such as the Connor-Davidson Resilience Scale (CD-RISC), Brief Resilience Scale (BRS), and Mental Toughness Questionnaire (MTQ). Detailed characteristics are presented in Table 1.

**Table 1:** Characteristics of the included studies

Number	Study (Author, Year)	Study Design	Country	Sample Size	Age/Population	Intervention method	Control Group Description	Resilience Measure	Outcome Data (Mean ± SD)
1	Terkes et al., 2023 [27]	RCT	Turkey	Total: 70 Monitoring Group: 35 Unsupervised group: 35	> 60 Diabetes	Online monitoring exercise, 3 times per week, for 6 weeks	Minimal intervention control: unsupervised exercise	Brief Resilience Scale	Monitoring Group: Pretest: 21.03 ± 7.86 Post-test: 23.54 ± 4.98 Unsupervised group: Pretest: 20.78 (7.05) Post-test: 21.71 (5.79)
2	Borrega-Mouquinho et al., 2021 [23]	RCT	Spain	Total: 67 HIIT group: 36 MIT Group: 31	18–60 Healthy	High-Intensity Interval Training + Moderate-intensity continuous training (6 days per week, 40 min per session, for 6 weeks)	No blank control	Connor-Davidson Resilience Scale	HIIT group: Pretest: 31.14 (5.2) Post-test: 32.04 (5.93) MIT group: Pretest: 29.29 (7.27) Post-test: 32.06 (6.16)
3	Moore et al., 2021 [28]	RCT	Australia	Total: 283; Experimental group: 142 Control group: 141	< 18 Healthy	10-week martial arts training + psychological education	No intervention	Child and Youth Resilience Measure	experimental group Pretest: 2.89 (0.52) Post-test: 2.90 (0.56) control group Pretest: 2.90 (0.56) Post-test: 2.62 (0.61)
4	Glaser et al., 2022 [29]	Quasi-experimental	Israel	Total: 56 Experimental group: 27 Control group: 29	< 18 Healthy	Online Sports Activity Health + "Heart-to-Heart" Dialogue (Weekly Online Exercise + "Heart-to-Heart" Dialogue, for approximately 3 months)	No intervention	Brief Resilience Scale	experimental group Pretest: 2.71 (0.40) Post-test: 3.45 (0.46) control group Pretest: 3.34 (0.57) Post-test: 3.35 (0.61)
5	Liu et al., 2025 [10, 12]	RCT	China	Total: 80 Experimental group: 40 Control group: 40	< 18 ADHD	60 min of aerobic exercise (once per week for 12 weeks)	No intervention	Connor-Davidson Resilience Scale	experimental group Pretest: 50.85 (24.37) Post-test: 56.50 (16.94) control group Pretest: 46.42 (20.27) Post-test: 43.90 (17.75)
6	Sarkissian et al., 2018 [30]	Single-group pre-post	America	Total: 30	< 18 Healthy	Kundalini Yoga Course(10 week)	No control group	Resilience Scale	experimental group Pretest: 122.30 (25.01) Post-test: 135.80 (26.24)
7	Koch et al., 2022 [31]	RCT	Germany	Total:120 Experimental group: 60 Control group: 60	18–60 Healthy	Yoga (primarily focused on breathing exercises called pranayama) (Once a week, for 1 h each time, for 6 months)	no yoga intervention	Resilience Scale	Intervention group: Pretest: 69.20 (14.85) Post-test: 73.80 (5.53) control group: Pretest: 69.07 (12.78) Post-test: 66.78 (8.48)
8	Friedman et al. 2022 [24]	Single-group pre-post	America	Total: 55 Weactive: 31 Wemindful: 24	18–60 Healthy	WeActive (aerobic strength training) and WeMindful (mindfulness yoga) for 8 weeks	No blank control	Connor-Davidson Resilience Scale	WeActive Pretest: 26.46 (6.14) Post-test: 27.23 (8.18) WeMindful Pretest: 24.5 (5.91) Post-test: 26.08 (5.22)
9	Kukihara et al. 2020 [22]	RCT	Japan	Total: Exercise group: 22 Control group: 18 Yoga: 15	> 60 Healthy	1. Exercise intervention (50 min of aerobic exercise per week, for 12 weeks) 2. Mindfulness Yoga Intervention (50 min of yoga per week, for 12 weeks)	No intervention	Resilience scale	Exercise group: 64.42 (16.43) Mindfulness Yoga Group: 64.20 (15.90) Control group: 48.77 (17.22)
10	Eyre et al., 2017 [25]	RCT	America	Total: 79 KY group: 38, MET group: 41	> 60 MCI	Kundalini Yoga (12 weeks)	No blank control	Connor-Davidson Resilience Scale	ky group: Pretest: 75.08 (13.66) Post-test: 78.41 (10.56) MET group: Pretest: 72.29 (14.38) Post-test: 74.10 (14.90)

### Quality assessment of included studies

The results of the methodological quality and risk of bias assessment using the modified Downs and Black checklist are summarized in Table 2. Total scores ranged from 15 to 31, with a mean score of 22.5.

**Table 2:** Methodological quality assessment of included studies using the downs and black checklist

Study	1	2	3	4	5	6	7	8	9	10	11	12	13	14	15	16	17	18	19	20	21	22	23	24	25	26	27	Total
Terkes et al., 2023 [27]	1	1	1	1	0	1	1	0	1	0	0	0	0	0	0	0	1	1	0	1	1	1	1	1	0	1	5	**20 **
Borrega-Mouquinho et al., 2021 [23]	1	1	1	1	2	1	1	0	1	1	0	0	1	0	0	1	1	1	1	1	1	1	1	0	1	1	5	**26 **
Moore et al., 2021 [28]	1	1	1	1	2	1	1	0	0	1	0	0	1	0	0	1	1	1	1	1	1	1	1	0	1	1	5	**25**
Glaser et al., 2022 [29]	1	1	1	1	2	1	1	0	0	1	0	0	1	0	0	1	1	1	1	1	1	1	0	0	1	1	3	**22**
Liu et al., 2025 [10, 12]	1	1	1	1	2	1	1	1	1	1	1	1	1	0	1	1	1	1	1	1	1	1	1	1	1	1	5	**31**
Sarkissian et al., 2018 [30]	1	1	1	1	0	1	1	0	1	1	1	0	1	0	0	1	1	1	0	1	0	1	0	0	0	0	0	**15**
Koch et al., 2022 [31]	1	1	1	1	2	1	1	0	1	1	1	1	1	0	0	1	1	1	1	1	1	1	1	0	1	1	5	**28**
Friedman et al. (2022) [24]	1	1	1	1	2	1	1	0	0	1	0	0	1	0	0	1	1	1	0	1	1	1	0	0	1	0	3	**20**
Kukihara et al. (2020) [22]	1	1	1	1	2	1	1	1	0	1	0	0	1	0	0	1	1	1	1	1	1	1	1	0	1	0	3	**23**
Eyre et al., 2017 [25]	1	1	1	1	1	1	1	1	0	1	0	0	0	0	1	1	1	1	0	1	1	1	1	0	1	0	0	**18**

Downs and Black [18] checklist: 27 items, total score 0–32. Items 1–10 = reporting,11–13 = external validity; 14–20 = internal validity (bias); 21–26 = internal validity (confounding); 27 = statistical power (0–5). Most items scored 0/1; item 5 scored 0–2; item 27 scored 0–5. Higher scores = better quality

### Meta-analysis: overall effect

**Fig. 2:**
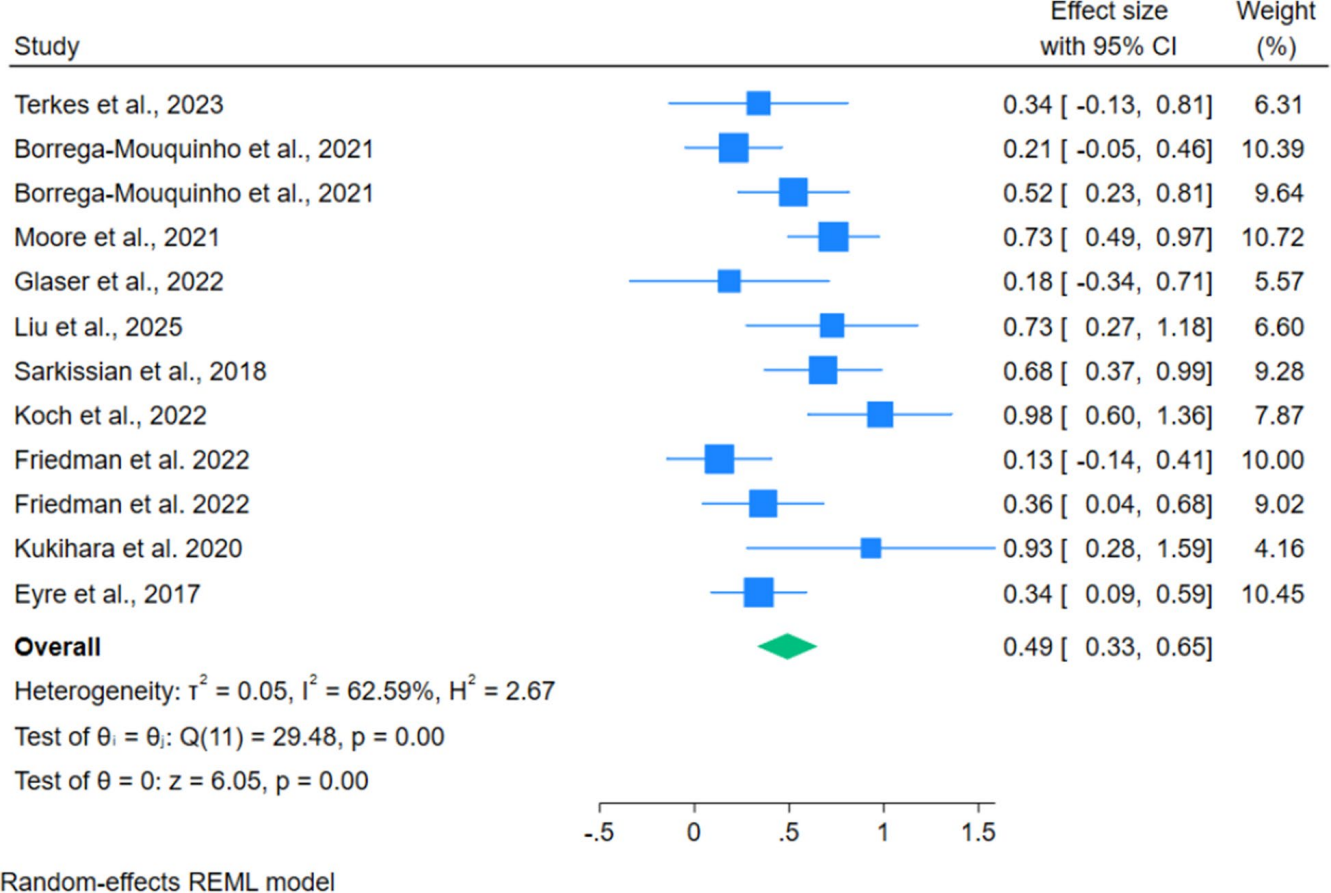
Forest plot of the effect of exercise interventions on psychological resilience compared to control conditions

The meta-analysis, conducted using a random-effects model on 10 studies comprising 12 experimental groups, yielded a significant positive overall effect of exercise intervention on psychological resilience (SMD = 0.49, 95% CI: 0.33 to 0.65, z = 6.05, p < 0.001). However, substantial between-study heterogeneity was observed (I^2^ = 62.59%, τ^2^ = 0.05), with a significant heterogeneity test (Q(11) = 29.48, p < 0.001), indicating that the variation in effect sizes stemmed not only from sampling error but also from genuine differences among studies. Individual study effect sizes ranged from 0.13 to 0.98, with the majority showing positive effects and only a few demonstrating non-significant effects. Study weights ranged from 4.16% to 10.72%, indicating a relatively balanced distribution that did not introduce substantial bias to the overall result. In summary, exercise intervention was effective in enhancing psychological resilience overall, but the effect was substantially influenced by study characteristics, suggesting the need to further explore potential moderators such as exercise type, intervention duration, and population features. The forest plot illustrating the individual and pooled effect sizes for the overall analysis is presented in Fig. 2.

### Subgroup analyses

#### Subgroup analysis by study design

**Fig. 3:**
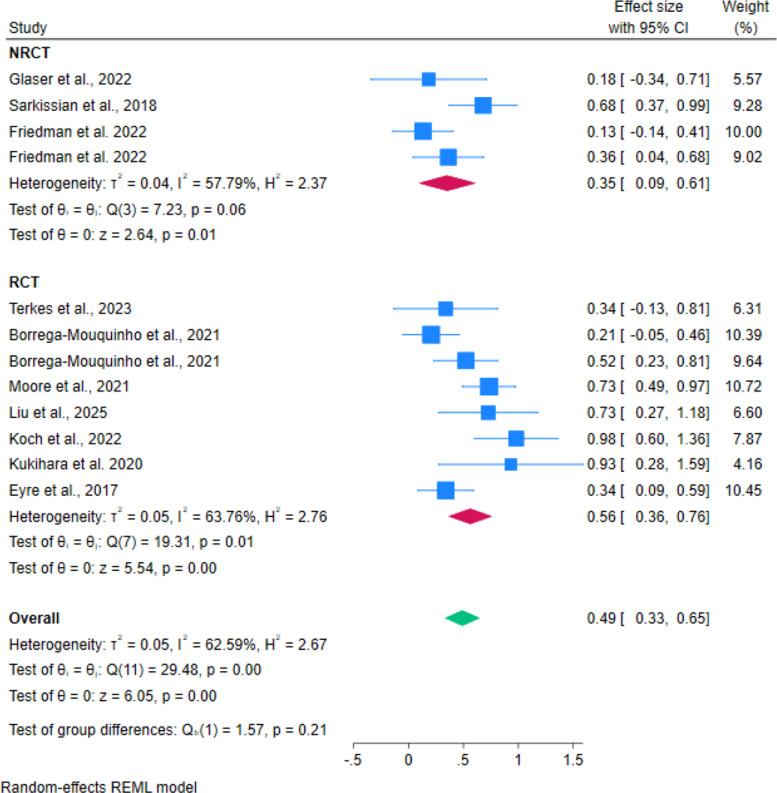
Forest plot of subgroup analysis by study design (RCTs vs. NRCTs)

Subgroup meta-analyses were performed based on the random-effects model. The overall pooled effect size was 0.49 (95% CI: 0.33 to 0.65, z = 6.05, *p* < 0.001), indicating a significant positive effect. Significant moderate to high heterogeneity persisted (I^2^ = 62.59%, τ^2^ = 0.05, Q(11) = 29.48, *p* < 0.001). In the analysis by study design, the pooled effect size for non-randomized controlled trials (NRCTs) was 0.35 (95% CI: 0.09 to 0.61, z = 2.64, *p* = 0.01), which was statistically significant, with moderate within-subgroup heterogeneity (I^2^ = 57.79%). In contrast, randomized controlled trials (RCTs) showed a significant pooled effect size of 0.56 (95% CI: 0.36 to 0.76, z = 5.54, *p* < 0.001), albeit with moderate to high within-subgroup heterogeneity (I^2^ = 63.76%). The test for subgroup differences was not statistically significant (Qb(1) = 1.57, p = 0.21; Bonferroni-adjusted α = 0.017). Exercise intervention showed an overall positive effect. While evidence from RCTs appeared more robust, study design did not emerge as a statistically significant moderator, suggesting that factors other than design may contribute to the observed heterogeneity. The results of this subgroup analysis are displayed in Fig. 3.

### Subgroup analysis by age

**Fig. 4:**
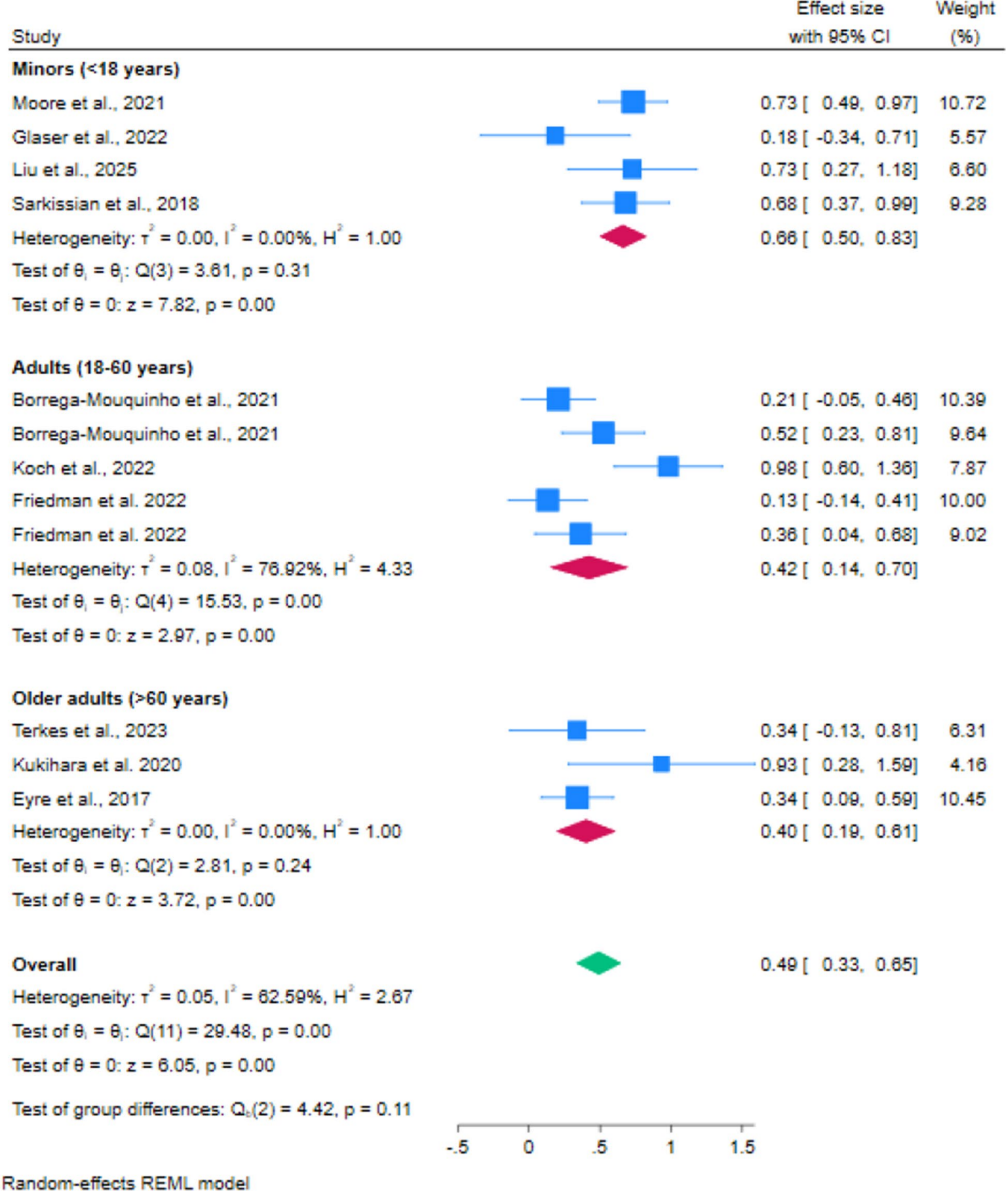
Forest plot of subgroup analysis by age group (minors, adults, older adults)

Subgroup analysis by age (minors, adults, older adults) was conducted using the random-effects model. Subgroup analysis by age group was performed using the random-effects model. For minors, the pooled effect size was 0.66 (95% CI: 0.50 to 0.83, z = 7.82, *p* < 0.001) with no within-subgroup heterogeneity (I^2^ = 0.00%), indicating a consistent and stable effect in this age group. For adults, the pooled effect size was 0.42 (95% CI: 0.14 to 0.70, z = 2.97, *p* = 0.00), which was significant but accompanied by high heterogeneity (I^2^ = 76.92%), suggesting the effect may be influenced by other factors. For older adults, the pooled effect size was 0.40 (95% CI: 0.19 to 0.61, z = 3.72, *p* = 0.00), also significant with no within-subgroup heterogeneity (I^2^ = 0.00%), indicating a consistent and stable effect in this population. The test for subgroup differences across age groups was not statistically significant (Qb(2) = 4.42, *p* = 0.11; Bonferroni-adjusted α = 0.017), indicating that age was not a significant moderator in this analysis. In summary, exercise intervention enhanced psychological resilience across all age groups, with the most consistent effects observed in minors and older adults, while greater variability was seen in adults. Fig. 4 presents the forest plot for the subgroup analysis based on participant age.

#### Subgroup analysis by intervention duration

**Fig. 5:**
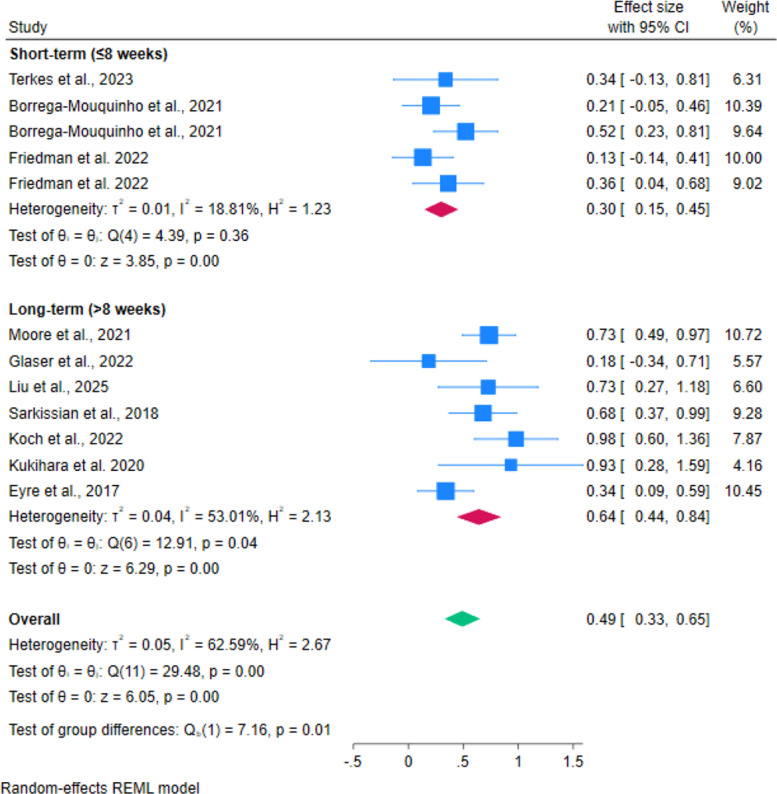
Forest plot of subgroup analysis by intervention duration (short-term vs. long-term)

Subgroup analysis based on intervention duration (long-term: > 8 weeks; short-term: ≤ 8 weeks) was performed using the random-effects model. Subgroup analysis based on intervention duration was performed using the random-effects model. The pooled effect size for short-term interventions was 0.30 (95% CI: 0.15 to 0.45, z = 3.85, *p* = 0.00) with low heterogeneity (I^2^ = 18.81%). In contrast, long-term interventions showed a larger effect size of 0.64 (95% CI: 0.44 to 0.84, z = 6.29, *p* < 0.001), albeit with moderate heterogeneity (I^2^ = 53.01%). The test for subgroup differences was statistically significant (Qb(1) = 7.16, *p* = 0.01) and survived Bonferroni correction for multiple subgroup tests (α < sub > adjusted </sub > = 0.017). Intervention duration therefore emerged as a statistically significant moderator. In conclusion, exercise interventions effectively enhanced psychological resilience, with long-term interventions yielding significantly greater benefits than short-term ones. The effect of long-term interventions showed moderate variability across studies, indicating the presence of other potential moderating variables. Fig. 5 visually summarizes the comparison of effect sizes between short-term and long-term interventions.

### Reporting bias and sensitivity analysis

#### Reporting bias

**Fig. 6:**
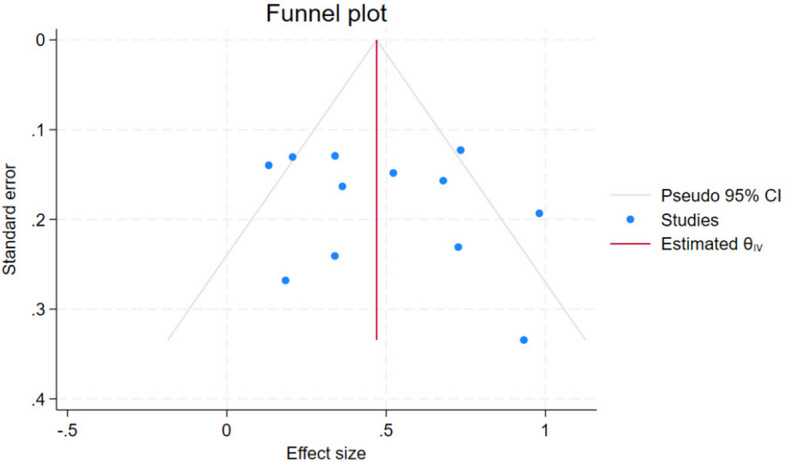
Funnel plot of standard error by standardized mean difference to assess publication bias

As shown in Fig. 6, the funnel plot appeared roughly symmetrical overall, with most studies clustered around the mean effect size. A single outlier study with a large effect size and relatively high standard error was observed in the lower right quadrant. Given the limited number of included studies (12 effect sizes), reliable detection of subtle deviation from perfect symmetry remains challenging. Egger’s regression test results (bias coefficient = 1.33, SE = 1.488, z = 0.89, *p* = 0.373) indicated no statistically significant asymmetry in the funnel plot. Therefore, no substantial evidence of publication bias or small-study effects was detected in this meta-analysis.

### Sensitivity analysis

**Fig. 7:**
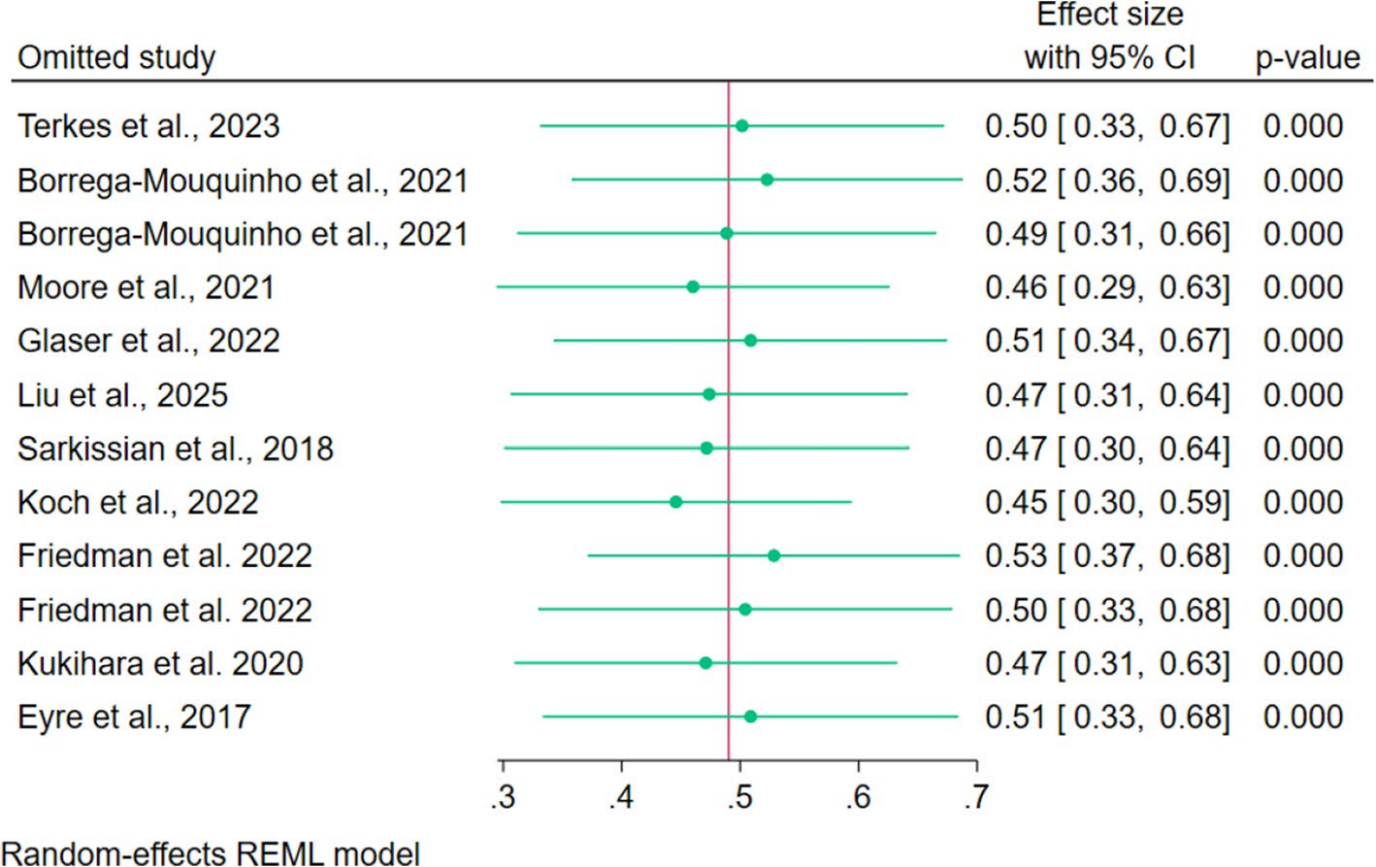
Leave-one-out sensitivity analysis of the included studies

To assess the robustness of the pooled effect size, a leave-one-out sensitivity analysis was conducted. The recalculated pooled standardized mean difference (SMD) ranged from 0.45 to 0.53 after iteratively removing each individual study, with the lower bound of the 95% confidence interval for all estimates remaining above zero. This indicates that the core finding of a significant positive effect of exercise intervention on psychological resilience (SMD = 0.49, 95% CI: 0.33–0.65) was not driven by any single study and demonstrates excellent robustness as shown in Fig. 7.

### Sensitivity analysis excluding combined interventions

**Fig. 8:**
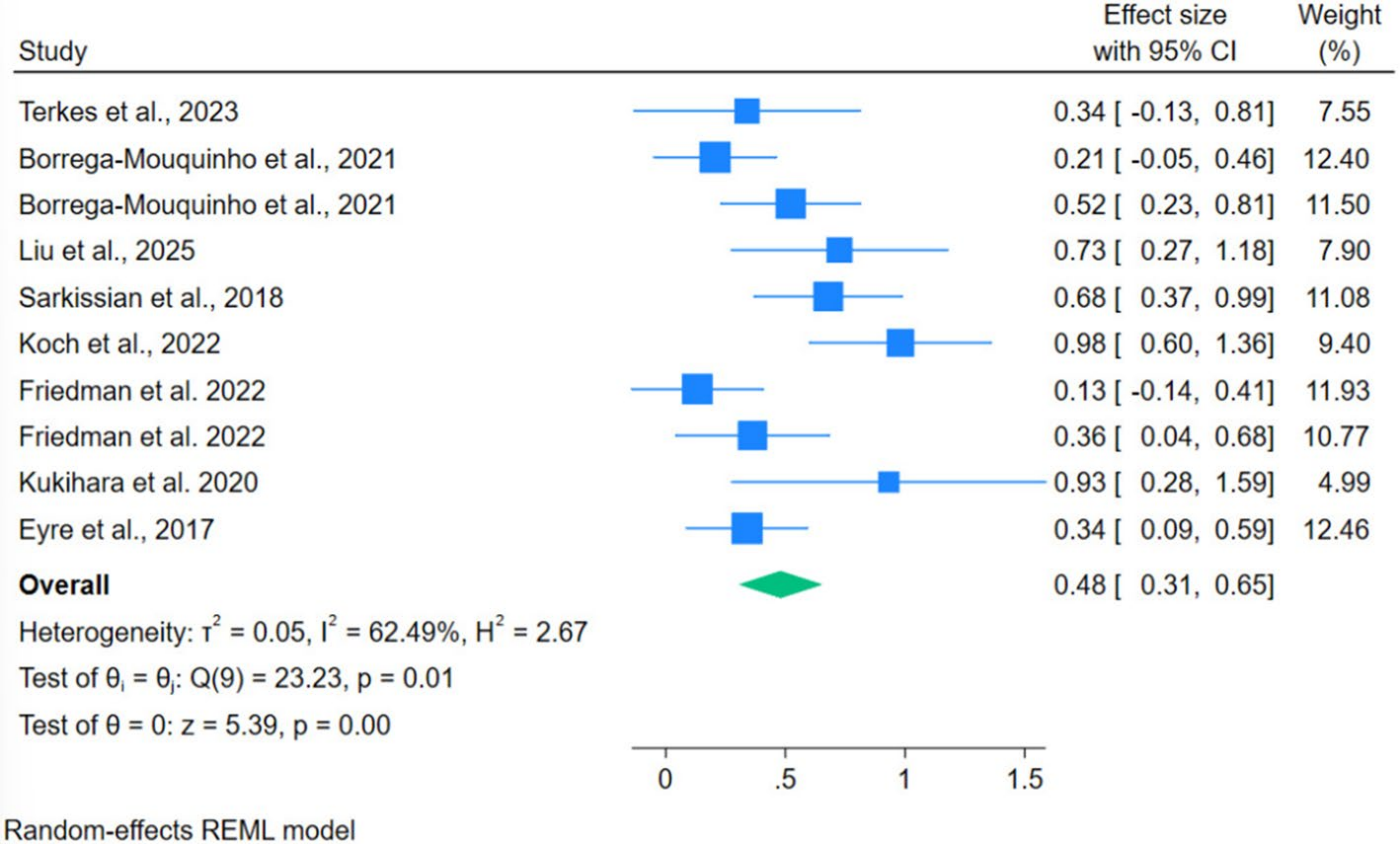
Forest plot of sensitivity analysis excluding combined exercise-psychological interventions [28, 29]

The forest plot for this sensitivity analysis is shown in Fig. 8. After excluding the two combined‑intervention studies [28, 29], the pooled standardized mean difference was 0.48 (95% CI: 0.31 to 0.65, z = 5.39, *p* < 0.001), with moderate to high heterogeneity (I^2^ = 62.49%, τ^2^ = 0.05). This effect size is nearly identical to that of the primary analysis (SMD = 0.49) and remains statistically significant, confirming that the positive effect of exercise on psychological resilience is robust and not an artefact of including combined interventions. A sensitivity analysis restricted to the eight effect sizes from studies using the CD‑RISC scale yielded a pooled SMD of 0.27 (95% CI: 0.13–0.42, *p* < 0.001, I^2^ = 46.2%), indicating that measurement tool diversity did not eliminate the significant effect.

To examine whether the use of different resilience measures influenced the results, we restricted the analysis to studies that used the CD‑RISC scale. The pooled SMD was 0.34 (95% CI: 0.20 to 0.48, z = 4.72, *p* < 0.001), with low heterogeneity (I^2^ = 27.65%) (Supplementary Figure S1).

### Pre-post correlation sensitivity analysis

A sensitivity analysis was conducted to evaluate the impact of the assumed pre-post correlation coefficient on the pooled effect size. When assuming a correlation of 0.50, the pooled SMD was 0.46 (95% CI: 0.29 to 0.63, z = 5.31, *p* < 0.001) with moderate heterogeneity (I^2^ = 56.97%). When assuming a correlation of 0.90, the pooled SMD was 0.63 (95% CI: 0.45 to 0.82, z = 6.73, *p* < 0.001) with high heterogeneity (I^2^ = 85.76%). In both scenarios, the overall effect remained statistically significant, and the magnitude of the effect was consistent with the primary analysis (SMD = 0.49). This confirms that our core conclusion regarding the positive effect of exercise on psychological resilience is robust to variations in the assumed pre-post correlation coefficient. The forest plots for this sensitivity analysis are presented in Supplementary Figures S2 and S3.

## Discussion

### Key findings and their implications

This meta-analysis, by searching five core databases and including 10 high-quality intervention studies, provides synthesized evidence by first quantitatively validating the core association between exercise interventions and enhanced psychological resilience, along with key moderating characteristics, across the full age spectrum. The central finding is a significant positive effect of exercise intervention on resilience (pooled effect size = 0.49, 95% CI [0.33, 0.65], *p* < 0.001). The absence of significant publication bias, as indicated by funnel plot symmetry and Egger’s test (bias coefficient = 1.33, SE = 1.488, z = 0.89, *p* = 0.373), confirms exercise as a reliable, cross-age intervention for boosting resilience. This aligns with the WHO framework, which advocates physical activity for population-wide mental health [32]. The subgroup analysis by intervention duration yielded a nominally significant result (Qb = 4.63, *p* = 0.03), though it did not survive Bonferroni correction for multiple subgroup comparisons (α < sub > adjusted </sub > = 0.017). Long-term interventions (> 8 weeks,SMD = 0.58) produced a substantially larger effect size than short-term interventions (≤ 8 weeks; SMD = 0.24), suggesting a pattern consistent with a duration–response relationship. This duration effect may partly reflect higher cumulative adherence in longer programs rather than duration per se, as sustained participation facilitates adaptation to the exercise regimen. Disentangling these two mechanisms requires future trials with systematic adherence tracking. This finding suggests that fostering resilience requires sustained exercise to build stable psychophysiological synergy. While not statistically significant (p = 0.21), the larger effect in RCTs underscores the importance of rigorous methodology, though caution is warranted. While age was not a significant moderator overall (Qb = 4.42, *p* = 0.11), the contrast between the consistent effect in minors (I^2^ = 0.00%) and the applicability across all age groups provides empirical grounds for personalizing interventions.

The core conclusions echo and complement several high-impact studies. Qiu et al. [6] confirmed a significant positive correlation between physical activity and resilience in students aged 12–25. Although RCTs in our analysis yielded a numerically larger effect size (SMD = 0.56) than non-randomized studies (SMD = 0.35), this subgroup difference was not statistically significant (*p* = 0.21). The lack of a significant difference may reflect insufficient statistical power rather than true equivalence. We therefore caution against interpreting study design as a direct moderator of intervention efficacy,rather, methodological rigor may influence effect size precision without necessarily reflecting differential intervention benefits.

Regarding the moderating role of intervention duration, our results contribute to a coherent evidence chain. Andermo et al. [17] found significant effects for 8–11 weeks of school-based physical activity in children/adolescents. Wu et al. [33] demonstrated that 12-week mind–body interventions were superior to shorter programs in older adults, indicating this duration-response pattern is not age-limited but may be influenced by baseline health and adherence.

Regarding age differences, the zero heterogeneity in minors (I^2^ = 0.00%) and older adults (I^2^ = 0.00%) is consistent with Belcher et al. [15]—the high plasticity of the adolescent brain and the relatively homogeneous lifestyle of older adults likely lead to more direct effects. Conversely, the high heterogeneity in adults (I^2^ = 76.92%) aligns with Wu et al.'s [33] observation that effects are influenced by comorbidities and adherence. This suggests age itself is not a direct moderator,rather, variations in neurodevelopmental stage and life stressors across age groups contribute to differential effect variability.

### In-depth analysis of heterogeneity sources

The overall heterogeneity was high (I^2^ = 62.59%), stemming primarily from three sources. First, exercise modalities and their underlying mechanisms varied substantially. Included studies encompassed online-supervised exercise, aerobic training, mindful yoga, HIIT, and resistance training. Kukihara et al. [22] found that mindful yoga and conventional exercise operated through distinct pathways, while Borrega-Mouquinho et al. [23] showed comparable effects between HIIT and moderate-intensity continuous training. Neurobiological mechanisms may also differ: resistance training modulates cortisol and IGF-1, whereas aerobic training affects BDNF [34, 35]. Marinelli et al. [36] reported that intervention characteristics varied widely—frequency (2–4 sessions/week), session duration (6–90 min), intervention length (5–20 weeks), and intensity (60%−70% 1RM or 8-12RM)—and found that interventions > 12 weeks yielded greater symptom reduction. As our analysis was not stratified by these factors, such variability likely contributed to heterogeneity.

Second, inconsistencies in resilience conceptualization and measurement were evident. Although all studies used standardized tools, these capture distinct facets: the CD-RISC assesses trait-like resilience [20], the BRS measures recovery ability [37], and other instruments tap related but distinct constructs. Denovan et al. [38] demonstrated that mental toughness, self-efficacy, and ego-resiliency share a core of Non-Cognitive Adaptive Resourcefulness but retain unique variance. Wolke et al. [39] emphasized that resilience can be operationalized as a trait, outcome, or process, and mixing these conceptualizations inflates heterogeneity. Van der Meer et al. [40] developed the RES to specifically capture self-confidence and self-efficacy, distinct from broader measures. Thus, using different instruments emphasizing different resilience aspects likely contributed to effect size variability, consistent with Qiu et al. [6]. This is further supported by our sensitivity analysis, which found that restricting the analysis to studies using the CD-RISC scale reduced heterogeneity to a low level (I^2^ = 27.65%).

Third, intervention implementation details were not systematically quantified. Andermo et al. [17] highlighted the importance of implementation fidelity, and Carmen et al. [41] found that interventions ≥ 3 times/week for ≥ 45 min/session yielded more stable effects. Consistent with Marinelli et al. [36], we observed considerable variability in the reporting of supervision, adherence, and baseline activity, further exacerbating heterogeneity.

Despite substantial heterogeneity, the pooled effect size remains meaningful. Under the random-effects model, SMD = 0.49 represents the average expected effect across diverse real-world scenarios [21]. Comprehensive sensitivity analyses confirmed excellent robustness of the core finding: leave-one-out analysis showed no single study drove the result,exclusion of combined exercise-psychological interventions yielded an almost identical effect size; restriction to CD-RISC-based studies maintained statistical significance; and varying the assumed pre-post correlation coefficient from 0.50 to 0.90 did not alter the direction or significance of the effect. Moderator analysis identified intervention duration as a significant factor (*p* = 0.01), explaining part of the variance. Residual heterogeneity does not invalidate our conclusion but highlights the need for future research to identify optimal intervention characteristics and adopt conceptually consistent measurement tools.Further exploration of exercise intensity or weekly frequency as moderators via meta-regression was precluded by inconsistent reporting of these parameters across the 10 included studies. Standardised reporting of FITT principles (frequency, intensity, time, type) in future trials would enable more granular moderator analyses.

### Strengths and innovations

This study demonstrates notable strengths and innovations. The literature search covered five core databases, adhered to PRISMA guidelines, and included 10 studies encompassing the full age spectrum. This comprehensive approach reduces selection bias and enhances the generalizability of findings compared to reviews focusing on single populations (e.g., [6, 42]. It is the first to quantitatively verify the statistically significant moderating effect of intervention duration after Bonferroni correction (p = 0.01, with > 8 weeks being optimal), thereby supplementing the qualitative conclusions of Andermo et al. [17]. Subgroup analyses showed that effects were most stable in minors and that RCTs yielded numerically larger effects, addressing the research need highlighted by Lan et al. [42]. The use of a random-effects model, along with funnel plot inspection and Egger's test to verify the absence of publication bias, and the application of the Downs and Black checklist for quality assessment, constitutes a more standardized methodology than the qualitative assessment employed by Wu et al. [33], resulting in more robust conclusions.

### Study limitations and future directions

This study has several limitations that should be considered when interpreting the findings. First, the analysis was based on only 10 studies, which limited the statistical power of subgroup analyses and precluded the use of meta-regression to explore potential sources of heterogeneity. Additionally, none of the included studies conducted post-intervention follow-up assessments; all outcomes were measured immediately after the intervention period. The durability of exercise-induced resilience enhancement therefore remains unknown. Future trials should incorporate follow-up assessments at 6 and 12 months post-intervention to determine whether resilience gains are sustained, decay, or require ongoing exercise participation to maintain. Moreover, the literature search was restricted to English and Chinese publications, which may have introduced language bias by excluding studies published in other languages (e.g., Spanish, German, French). The small number of studies also reduced the reliability of Egger’s test for detecting publication bias, as its statistical power is limited when fewer than 20 studies are included. Therefore, while no significant publication bias was detected, this result should be interpreted with caution. As new trials on exercise and psychological resilience are published, future updates of this meta-analysis will be able to confirm these findings and enable more robust moderator analyses. It included only 10 studies, some with small subgroup samples. Analyses were not stratified by exercise type or intensity, and potential confounders such as social support were not accounted for. The lack of long-term follow-up data precludes conclusions regarding effect durability. Furthermore, reliance on self-reported measures and the inclusion of some non-randomized controlled trials introduce potential biases. Finally, while we conducted a sensitivity analysis excluding combined exercise‑psychological interventions to isolate the effect of exercise alone, the small number of such studies (*n* = 2) precluded a formal subgroup comparison. The fact that the pooled effect remained significant after their exclusion (SMD = 0.48) supports the robustness of our conclusion, but does not rule out potential synergy between exercise and psychological components. Future trials should directly compare pure exercise versus combined interventions.Similarly, the effect remained significant (SMD = 0.34) when only CD‑RISC‑based studies were analysed, suggesting that the choice of resilience scale does not alter the main conclusion.

Future research should expand the sample size by including special populations, conduct more refined analyses of moderating variables to identify optimal exercise modalities and dosages, extend follow-up periods to at least one year, and optimize study design and measurement methods—for instance, by employing unified assessment tools combined with objective indicators. In practice, tailored interventions should be promoted across populations: integrate structured aerobic exercise into school-based mental health education, focus on adherence-enhancing strategies for adults, design low-intensity aerobic programs combined with social support for older adults, incorporate long-term exercise into rehabilitation plans for relevant patient groups, and extend "exercise for resilience" health promotion initiatives to the general public.

## Conclusion

This systematic review and meta-analysis indicates that exercise interventions have a moderate positive effect on enhancing psychological resilience (SMD = 0.49), with long-term programs (> 8 weeks) yielding significantly larger benefits than short-term ones. These findings were consistent across minors, adults, and older adults. However, the current evidence base is limited by a small number of studies, moderate to high heterogeneity, and incomplete reporting of participant characteristics (e.g., health status, baseline activity levels). These limitations preclude firm recommendations for specific populations or clinical settings at this stage. The observed statistically significant moderating role of intervention duration provides robust guidance for designing structured, long-term exercise programs, but personalization will require future trials that systematically vary exercise type, intensity, and dosage across well-characterized populations. Future research should also incorporate long-term follow-up assessments and objective measures of adherence and physiological adaptation. Pending further confirmatory evidence, structured long-term exercise interventions may be considered a promising component of mental health promotion in public health and educational contexts.

## Supplementary Information

Supplementary Material 1.

Supplementary Material 2.

## Data Availability

Data will be made available on reasonable request. Almost all the presented data are publicly available in the original research articles.
