# Supplementary material for: The effect of exercise interventions on enhancing psychological resilience: a systematic review and meta-analysis
Source: BMC Psychol. 2026 Jun 6;14:1167. doi: 10.1186/s40359-026-04940-5 (PMC13459223; doi:10.1186/s40359-026-04940-5)
Supplement: Supplementary file 1 [file 40359_2026_4940_MOESM1_ESM.pdf]

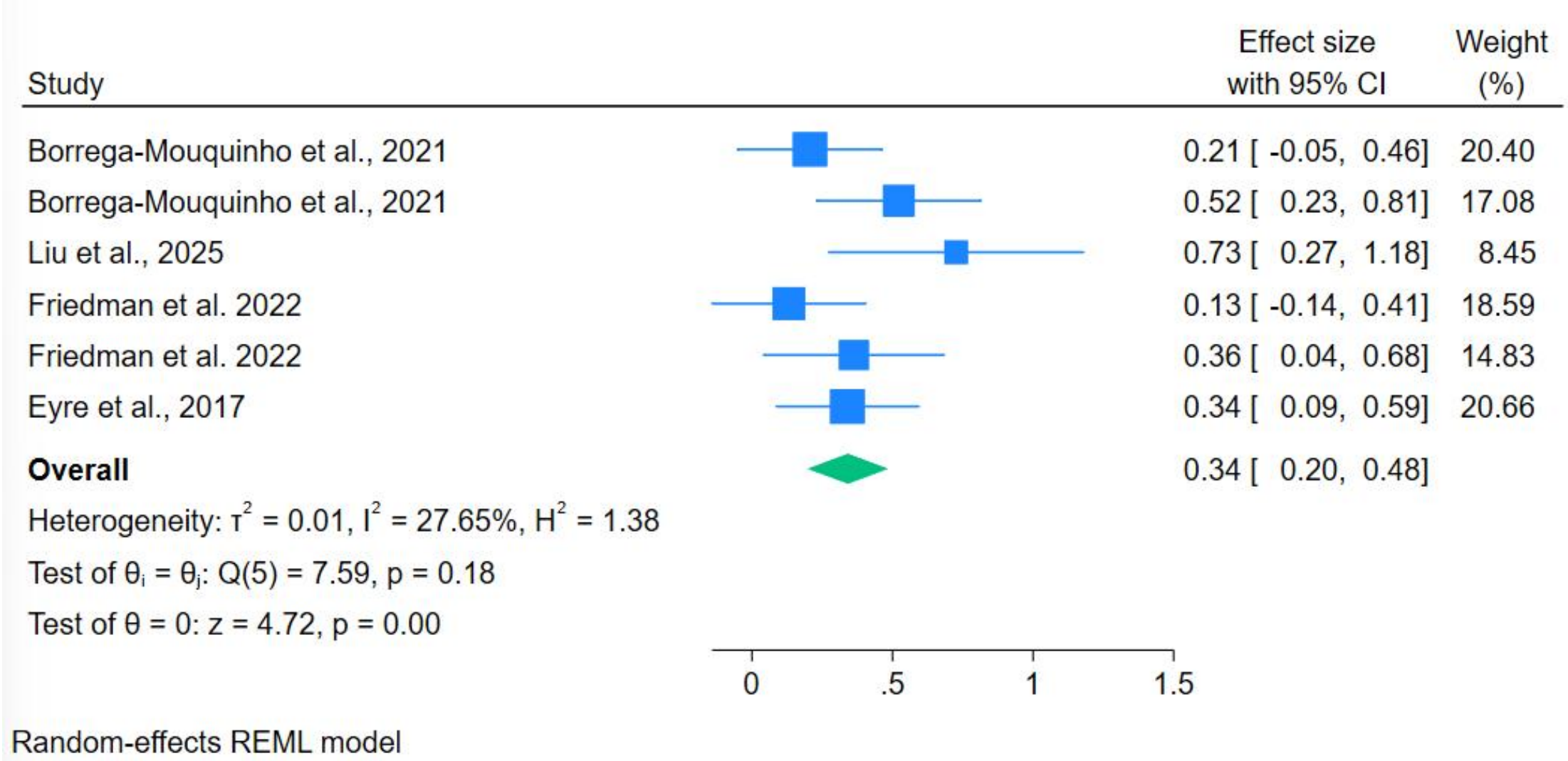

Supplementary Figure S1. Forest plot of sensitivity analysis restricted to studies using the CD-RISC scale. The analysis includes only studies that reported the Connor-Davidson Resilience Scale (CD-RISC).

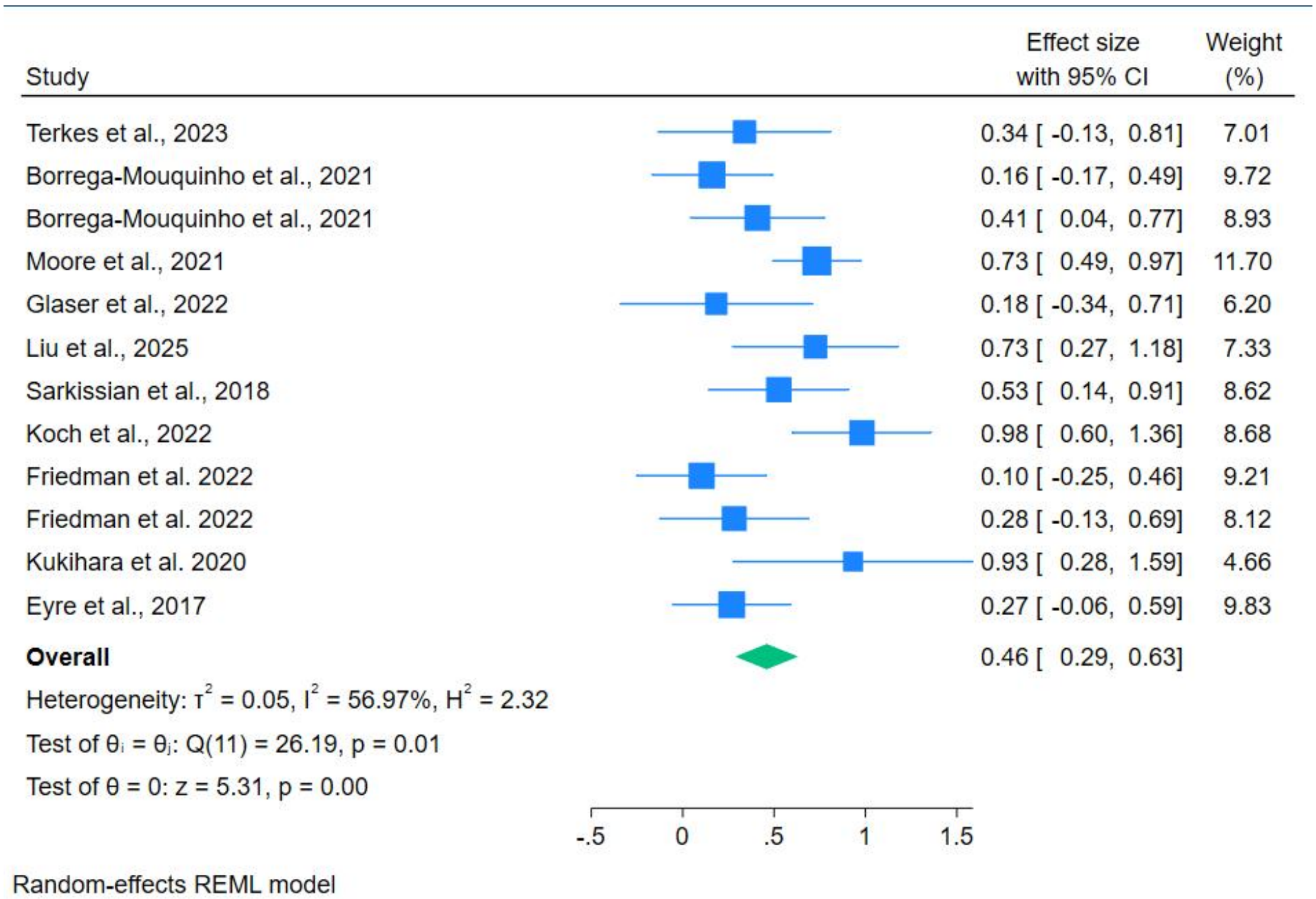

Supplementary Figure S2. Forest plot of sensitivity analysis assuming a pre-post correlation of r = 0.50.

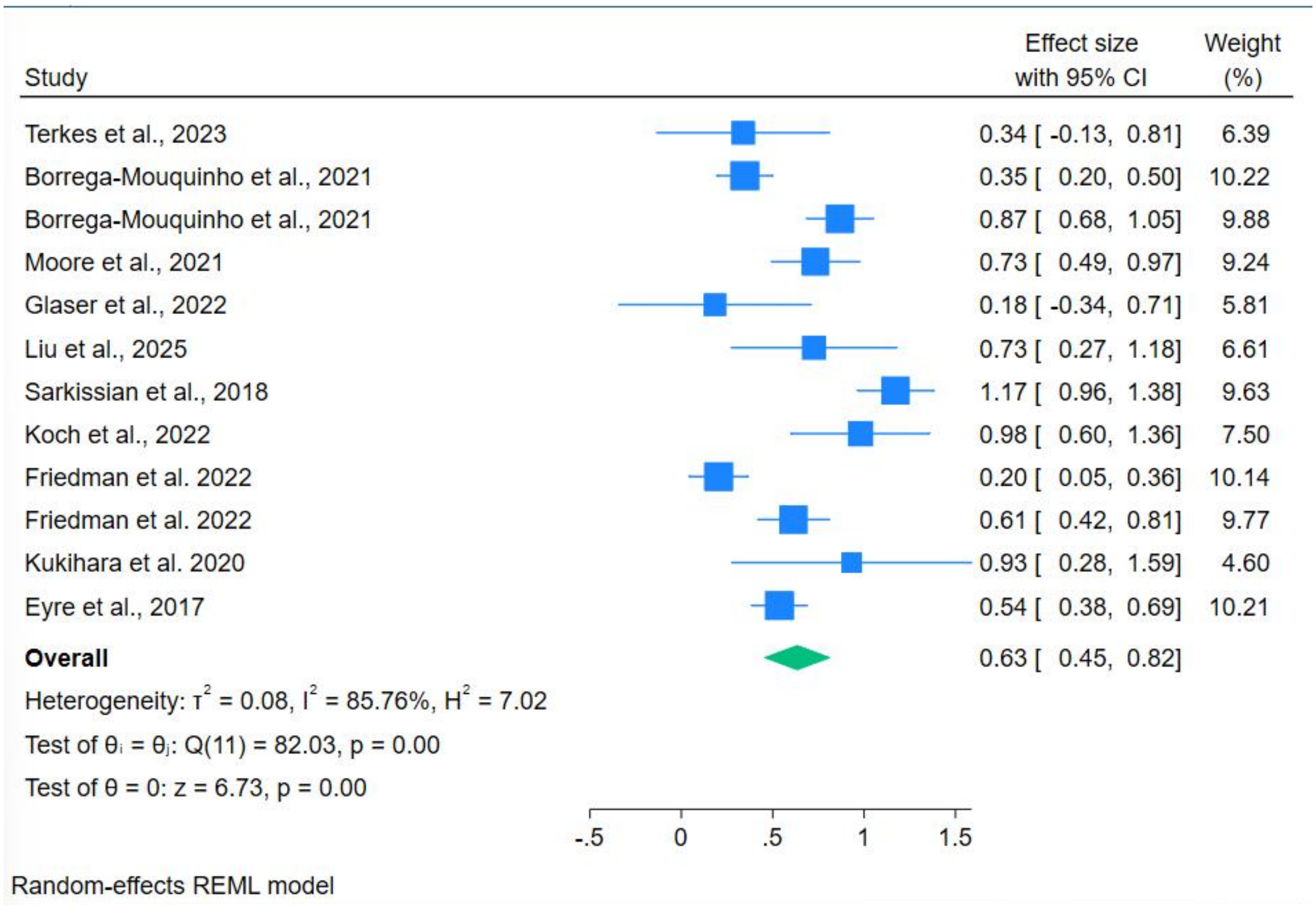

Supplementary Figure S3. Forest plot of sensitivity analysis assuming a pre - post correlation of r = 0.90.
